# The Role of Proteoglycans in Cancer Metastasis and Circulating Tumor Cell Analysis

**DOI:** 10.3389/fcell.2020.00749

**Published:** 2020-08-26

**Authors:** Theresa D. Ahrens, Sara R. Bang-Christensen, Amalie M. Jørgensen, Caroline Løppke, Charlotte B. Spliid, Nicolai T. Sand, Thomas M. Clausen, Ali Salanti, Mette Ø. Agerbæk

**Affiliations:** ^1^Centre for Medical Parasitology at Department of Immunology and Microbiology, Faculty of Health and Medical Sciences, University of Copenhagen and Department of Infectious Diseases, Copenhagen University Hospital, Copenhagen, Denmark; ^2^VarCT Diagnostics, Copenhagen, Denmark; ^3^Department of Cellular and Molecular Medicine, University of California, San Diego, La Jolla, CA, United States

**Keywords:** cancer, circulating tumor cells, diagnostic, glycosaminoglycan, liquid biopsy, metastasis, proteoglycan, VAR2CSA

## Abstract

Circulating tumor cells (CTCs) are accessible by liquid biopsies via an easy blood draw. They represent not only the primary tumor site, but also potential metastatic lesions, and could thus be an attractive supplement for cancer diagnostics. However, the analysis of rare CTCs in billions of normal blood cells is still technically challenging and novel specific CTC markers are needed. The formation of metastasis is a complex process supported by numerous molecular alterations, and thus novel CTC markers might be found by focusing on this process. One example of this is specific changes in the cancer cell glycocalyx, which is a network on the cell surface composed of carbohydrate structures. Proteoglycans are important glycocalyx components and consist of a protein core and covalently attached long glycosaminoglycan chains. A few CTC assays have already utilized proteoglycans for both enrichment and analysis of CTCs. Nonetheless, the biological function of proteoglycans on clinical CTCs has not been studied in detail so far. Therefore, the present review describes proteoglycan functions during the metastatic cascade to highlight their importance to CTCs. We also outline current approaches for CTC assays based on targeting proteoglycans by their protein cores or their glycosaminoglycan chains. Lastly, we briefly discuss important technical aspects, which should be considered for studying proteoglycans.

## Introduction

During cancer progression, metastatic spread occurs when cancer cells disseminate from the primary tumor and travel to a distant site to form a metastasis (Micalizzi et al., 2017). This can emerge through three major routes: the blood circulation, the lymphatic system, or via serosal or mucosal surfaces (Fidler, 1978). However, cancer cell dissemination through the blood is thought to be the main route of metastasis (Lambert et al., 2017), and the subset of cancer cells that have entered the blood circulation is named circulating tumor cells (CTCs). Intravasation of CTCs into the blood stream is believed to be one of the rate-limiting steps for metastasis formation and can occur through either an active invasion or passive shedding of cells from the tumor (Cavallaro and Christofori, 2001; Bockhorn et al., 2007; van Zijl et al., 2011). Only a minority of cancer cells reaching the blood circulation manages to survive shear stress, escape immune surveillance, avoid detachment-induced cell death, extravasate at the distant site, and finally establish a metastasis (Massague and Obenauf, 2016). Thus, the process of metastasis is both a complex and inefficient process (van Zijl et al., 2011; Reymond et al., 2013).

In addition to representing the primary tumor, CTCs have also been shown to exit from metastatic lesions (Kim et al., 2009). Such cells have the potential to travel back to the primary tumor site (called tumor self-seeding) or create another metastasis (Mentis et al., 2020). Therefore, CTCs could represent both the primary tumor and potential metastatic lesions (Kim et al., 2009), making CTC analyses highly relevant even years after surgical removal of the primary tumor. Hence, CTC analyses could provide important information about disease progression and relapse. Furthermore, molecular analyses of CTCs including mutational profiling, could provide the basis for personalized therapies in the future (Greene et al., 2012). Thus, CTCs are currently evaluated for clinical diagnostics. However, CTC analysis remains technically challenging, not only due to the rarity of CTCs among billions of normal blood cells, but also due to their inherent high degree of cellular plasticity complicating the choice of detection markers (Alix-Panabieres et al., 2017). Accordingly, CTC enrichment and detection strategies must be based on highly specific biomarkers to achieve the necessary assay specificity and sensitivity. Moreover, targeting a broader CTC population would be beneficial to ensure that the liquid biopsy better reflects the heterogenic cancer cell population.

**FIGURE 1:**
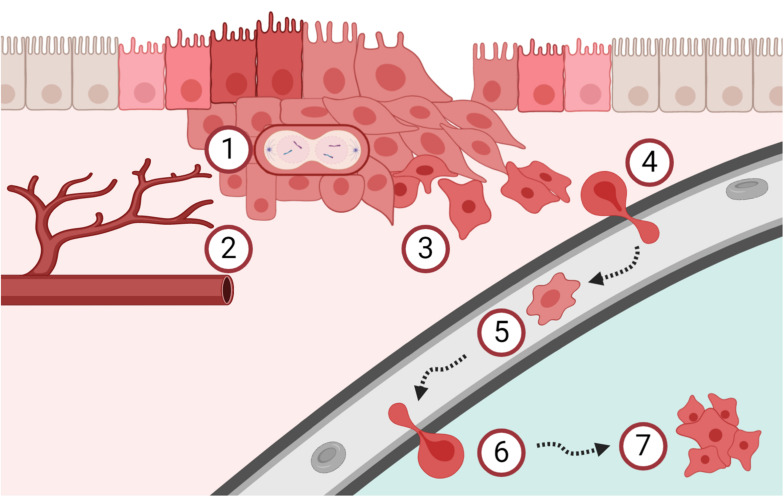
Schematic overview of the metastatic cascade. **(1)** Cancer cells start to proliferate uncontrolled and **(2)** tumor angiogenesis is mandatory to support continued tumor growth, already early during carcinogenesis. **(3)** The process of epithelial-mesenchymal transition increases the migration and invasion capacity of cancer cells. **(4)** Cancer cells intravasate into the blood circulation and **(5)** are then called circulating tumor cells (CTCs). CTCs are easily accessible by liquid biopsies and are currently investigated as tool for cancer diagnostics and surveillance. **(6)** A subpopulation of these CTCs has the potential to extravasate and **(7)** form metastasis in secondary organs. Clearly, the metastatic process is very complex and many of these steps are interconnected. Please refer to the main text for details and references.

As a part of discovering novel CTC targets, many strategies focus on proteins known to play an active role in metastatic seeding. Although solid tumors differ in their metastatic patterns, they share certain mechanistic similarities for metastasis formation, which are summarized as the metastatic cascade (Figure 1) (de Groot et al., 2017; Lambert et al., 2017; Riggi et al., 2018). Notably, the majority of steps in this process concerns the interaction between the cancer cells and the surrounding extracellular matrix (ECM). Therefore, novel clinically relevant CTC targets may be discovered within the pericellular layer called the glycocalyx.

**FIGURE 2:**
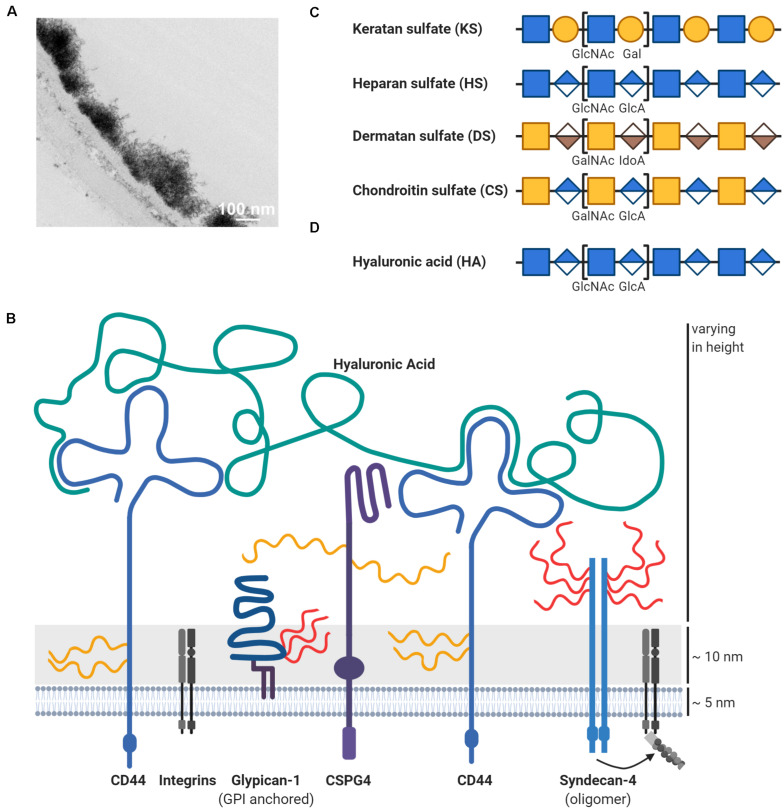
Schematic overview of proteoglycans in the glycocalyx. **(A)** The glycocalyx is a dense network of carbohydrates on the cell surface, here exemplified on top of the endothelium. This transmission electron microscopic image with lanthanum nitrate staining was reused from Okada et al. (2017) under CC BY 4.0 license. **(B)** The thick carbohydrate layer on the cell surface extends beyond the length of membrane proteins like integrins. One important glycocalyx component are proteoglycans, which consist of a protein core (blue) and covalently attached glycosaminoglycans such as heparan sulfate (HS; in red) or chondroitin sulfate (CS; in yellow). Depicted are some proteoglycans, which are mentioned and discussed throughout the review like chondroitin sulfate proteoglycan 4 (CSPG4). Hyaluronic acid (HA; in green) is another important glycosaminoglycan component in the glycocalyx, but is distinct through the lack of a protein core. Hyaluronic acid is attached to the cell surface via interactions with its receptors like CD44, which is itself a proteoglycan. Other glycocalyx components like glycoproteins are not shown due to abstractification. Depiction of the disaccharides units for **(C)** glycosaminoglycans attached to proteoglycans (keratan sulfate/KS, heparan sulfate/HS, dermatan sulfate/DS, and chondroitin sulfate/CS) and **(D)** of hyaluronic acid (HA), which is non-covalently attached to its receptors. Glycosaminoglycans can be subjected to further modifications, such as sulfation or epimerization, which is not shown for simplicity. Please refer to the main text for details and references.

The glycocalyx is a thick network of carbohydrates bound to glycoproteins, glycolipids, and proteoglycans (Figure 2A; reused from Okada et al., 2017). It is present on cell surfaces throughout the entire human body and constitutes a physical barrier between the cell and the surrounding microenvironment (Tarbell and Cancel, 2016). The glycocalyx plays a crucial role for receptor–ligand interactions of cancer cells and their surroundings, enabling migration as well as intra- and extravasation. Furthermore, the composition of the glycocalyx is thought to influence the transportation and survival of CTCs in the bloodstream (Mitchell and King, 2014). However, very few studies have investigated the glycocalyx of CTCs. At present, it is best studied in endothelial cells, where it serves as a physical and electrostatic barrier as well as a mechanotransducer toward other cells, the extracellular matrix (ECM), or shear forces of the blood (Reitsma et al., 2007; Butler and Bhatnagar, 2019). To convey signaling, growth factors, chemokines, and other interaction partners have to navigate through this dense structure. The glycocalyx, which extends beyond the length of most surface receptors, has a dual role in signaling by creating a physical hindrance for ligand receptor interactions or by promoting binding once interaction partners are in close proximity to each other (Kuo et al., 2018). Moreover, certain glycocalyx components are involved in chemokine storage and oligomerization, which strongly modulates their signaling strength (Salanga and Handel, 2011). Therefore, glycocalyx changes can have various effects on cellular behavior and, not surprisingly, cancer cells show specific alterations in their glycocalyx.

The cancer cell glycocalyx is a highly dynamic structure from which most of the components have been linked to the acquisition of oncogenic phenotypes (Daniotti et al., 2015; Buffone and Weaver, 2020). For instance, aberrant glycosylations including hypersialylation support immune evasion mechanisms (Pearce and Läubli, 2016). Moreover, increased expression of bulky glycoproteins like mucin-1 has been linked to aggressive cancers (Paszek et al., 2014; Barnes et al., 2018) and associated with poor survival outcome in patients (Kufe, 2009). This might be explained by the bulkiness of the mucin-1 ectodomain shaped by numerous glycosylations, which facilitates integrin clustering, cell signaling, and cell proliferation (Paszek et al., 2014; Woods et al., 2017; Kuo et al., 2018). Naturally, mucin-1 is of interest as a target for therapy (Pillai et al., 2015), due to its high involvement in cancer. Likewise, CTCs were found to express high mucin-1 levels (Paszek et al., 2014), and mucin-1 has also been explored for CTC capture and detection (Muller et al., 2012; Strati et al., 2013; Schehr et al., 2016).

Another important component of the glycocalyx are proteoglycans (Figure 2B) with multiple implications in metastatic dissemination of cancer cells and tumor cell growth (Iozzo and Sanderson, 2011; Vitale et al., 2019). Proteoglycans can be secreted into the ECM or located intracellularly as well as on the cell surface either directly embedded in the plasma membrane or anchored by a glycosylphosphatidylinositol (GPI)-linker (Iozzo and Schaefer, 2015). Proteoglycans consist of two functional units: protein core and glycosaminoglycan (GAG) chains (Walimbe and Panitch, 2019). Most commonly, the assembly of GAG chains occur from a tetrasaccharide linker region covalently attached to serine residues within the protein core (Esko and Zhang, 1996). The GAG family is classified by their chemical composition and includes chondroitin sulfate (CS), dermatan sulfate (DS), keratan sulfate (KS), heparan sulfate (HS), and hyaluronic acid (HA) (Figures 2C,D) (Toole, 2004; Bulow and Hobert, 2006). In general, GAGs consist of long linear repeats of disaccharide units consisting of hexuronic acids and hexosamines. The hexuronic acid epimers comprise D-glucuronic acid (GlcA) for CS/HA and L-iduronic acid (IdoA) for DS, whereas the hexosamine units consist of an *N*-acetyl-D-glucosamine (GlcNAc) for HS/KS/HA, and an *N*-acetyl-D-galactosamine (GalNAc) for CS/DS (Schaefer and Schaefer, 2010; Ghiselli and Maccarana, 2016; Pomin and Mulloy, 2018). The structures of GAGs are extremely diverse, as their synthesis in the Golgi apparatus is not based on a precise template, but on a redundant network of enzymes that seems to be regulated based on tissue and cell types (Dick et al., 2012; Mikami and Kitagawa, 2013; Chen Y. H. et al., 2018).

Besides the variation in the monosaccharide composition, the molecular diversity of GAGs also results from varying polymer lengths and extensive post-translational modifications such as sulfations and epimerizations along the chain (Bulow and Hobert, 2006). GAG sulfation patterns often determine their biological function and serve as specific recognition motifs for a wide variety of growth factors, cytokines, chemokines, and pathogens (Xu and Esko, 2014; Mizumoto et al., 2015; Pinho and Reis, 2015). Therefore, alterations in the GAG composition of proteoglycans in cancers have received a lot of interest (Sweet et al., 1976; Chandrasekaran and Davidson, 1979; De Klerk et al., 1984). A well-studied example is the change in sulfation patterns of GAGs, which likely depends on the specific cancer type. Some studies have reported high expression of CS 4-*O*-sulfotransferases in both ovarian and breast cancers, while a study on cancerous lung tissues found elevated 6-*O*-sulfated CS, compared to nonmalignant tissue (Cooney et al., 2011; Oliveira-Ferrer et al., 2015; Li et al., 2017). Similarly, various HS sulfotransferases have been found upregulated in different cancers. These include 6-*O*-sulfotransferases in ovarian and colorectal cancer; 3-*O-*sulfotransferases in breast and pancreatic cancer; along with *N*-deacetylase and *N*-sulfotransferases in hepatocellular carcinomas (Tatrai et al., 2010; Song et al., 2011; Hatabe et al., 2013; Cole et al., 2014; Vijaya Kumar et al., 2014). Moreover, several studies have reported increases in CS quantity or in expression of CS polymerization genes in malignant tissues, suggesting that CS polymers are pro-tumorigenic (Momose et al., 2016; Li et al., 2017; Hou et al., 2019).

Interestingly, some proteoglycans may be modified with different GAG types simultaneously, as seen for syndecans, which can carry both CS/DS and HS, dependent on the structure of the ectodomain (Kokenyesi and Bernfield, 1994; Iozzo and Schaefer, 2015). Similarly, the HA-binding proteoglycan, versican, undergoes alternative splicing of exons encoding the GAG-attachment region resulting in altered GAG display. Notably, expression of distinct versican isotypes was shown to facilitate cancer progression in multiple cancer types (Dours-Zimmermann and Zimmermann, 1994; Theocharis et al., 2015; Zhangyuan et al., 2020). The protein cores of proteoglycans are, however, not just scaffolds for GAG extension, since they also directly bind ligands and mediate intracellular signaling in GAG-independent manners.

In contrast to the rest of the GAGs, HA stands out by not being covalently attached to a protein core (Figures 2B,D). Instead, HA is synthesized as an unmodified polysaccharide at the plasma membrane, where it is extruded from the cell surface and cleaved off into the ECM (Weigel and DeAngelis, 2007; Itano, 2008). In most cells, HA is an abundant structural component of the glycocalyx, where it interacts with receptors and surface proteoglycans via their hyaluronan-binding motifs and regulates the viscosity of the glycocalyx by its ability to retain water (Toole, 2001, 2004). Upon binding, HA triggers activation of a range of signaling pathways involved in cell proliferation, differentiation, motility, and adhesion, thereby influencing processes such as development, tissue homeostasis, and carcinogenesis (Turley et al., 2002; Toole, 2004; Liu et al., 2019). Although HA is extensively involved in cancer, it will not be discussed in detail throughout this review, as it is not considered a proteoglycan due to its lack of a protein core.

Altogether, proteoglycans compose a highly heterogeneous group of proteins that diverge by structural alterations of the protein core as well as by differences in their GAGs with regard to chain number, type, length, and post-translational modifications. Notably, proteoglycans are important integrators for cell signaling events with direct implications for carcinogenesis and cancer progression (Iozzo and Sanderson, 2011; Pinho and Reis, 2015; Nikitovic et al., 2018). In spite of this, the functions of proteoglycans and their GAGs in relation to CTCs are currently understudied. Therefore, this review will highlight examples of proteoglycans involved in the metastatic cascade with potential links to CTC biology. More specifically, we will discuss how proteoglycans play active roles in cancer cell proliferation, migration, survival, plasticity, and invasion with a dedicated focus on the function of both the protein core and the GAG chains. Finally, we provide an overview of proteoglycans that are currently evaluated for CTC technologies and briefly highlight some of the technical aspects to consider when studying proteoglycans.

## Proteoglycans in the Metastatic Cascade

### Cancer Cell Proliferation

Cancers show deregulation of their cell proliferation by various mechanisms. Proteoglycans can influence cell growth by interacting with growth factors, either via their core proteins or through their GAG chains, as observed for HS chains of heparan sulfate proteoglycans (HSPGs) (Knelson et al., 2014). Enzymes modifying GAGs may hence influence tumor growth (Morla, 2019) as demonstrated by sulfatases interfering with growth factor signaling through HS desulfation (Ai et al., 2003; Peterson et al., 2010; Vicente et al., 2015).

However, proteoglycans also influence tumor growth by GAG–independent mechanisms. The transmembrane chondroitin sulfate proteoglycan 4 (CSPG4) has been shown to positively regulate cancer cell proliferation in various cancer entities (Wang et al., 2011; Jamil et al., 2016; Hsu et al., 2018) and is currently under investigation for CTC capture and identification, as described in detail later. Studies have found that CSPG4 is involved in growth signaling by interaction through both its cytoplasmic domain and ectodomain (Yang et al., 2009; Stallcup, 2017). Through the extracellular part, CSPG4 potentiates the mitogen-activated protein kinase (MAPK) cascade by high-affinity, largely GAG-independent binding of growth factors, which are thus likely presented to their cognate receptors by CSPG4 (Nishiyama et al., 1996; Goretzki et al., 1999; Stallcup, 2002; Price et al., 2011). In glioma cell models, phosphorylation of the cytoplasmic CSPG4 domain induced proliferation, which was mediated by interaction with integrins (Makagiansar et al., 2007; Stallcup, 2017). Furthermore, CSPG4-mediated activation of one of the same integrins induced chemoresistance and survival in tumor cells (Chekenya et al., 2008). Thus, CSPG4 is an example of a proteoglycan positively regulating growth and survival via its protein domain.

**FIGURE 3:**
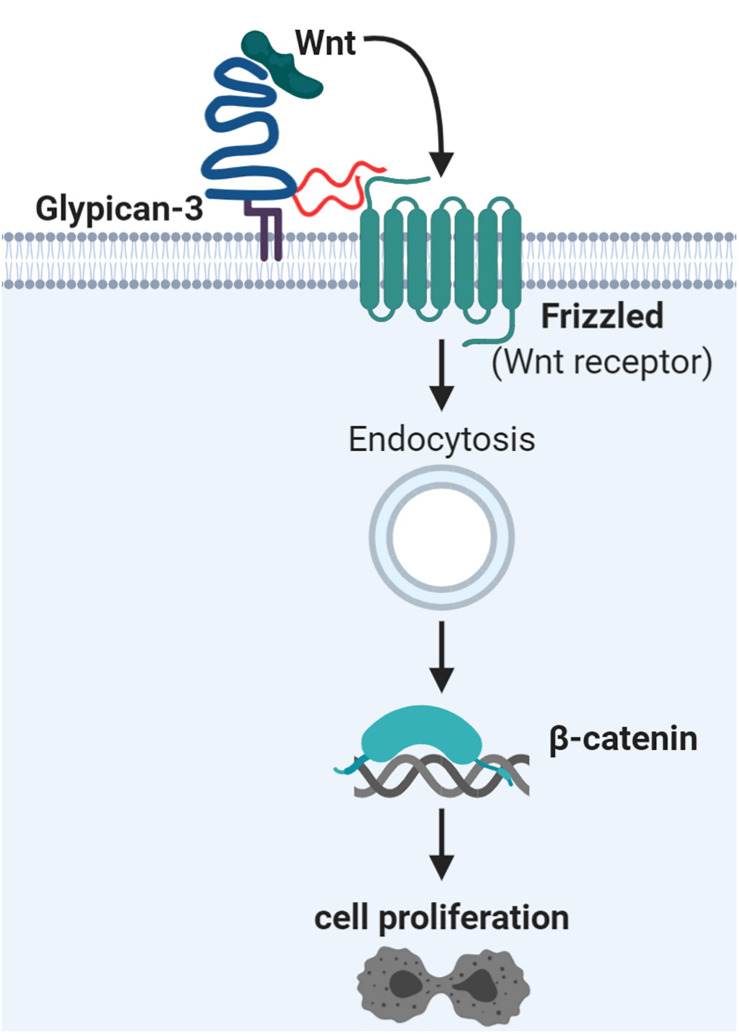
Glypican-3 signaling supports Wnt signaling and hepatocellular proliferation. Glypican-3 (shown in blue) can carry two glycosaminoglycan chains of heparan sulfate (HS; in red) or chondroitin sulfate (CS; not shown). It has been determined that these glycosaminoglycan chains are essential for interaction with Frizzled proteins, the Wnt receptors, but not for Wnt ligand binding. The ternary complex of glypican-3, Frizzled, and Wnt ligand becomes endocytosed as part of canonical Wnt signaling. This leads to nuclear accumulation of β-catenin and subsequent gene expression changes, stimulating cell proliferation. Details and references are given in the main text.

The proteoglycan glypican-3 (GPC3) has also been shown to increase cell proliferation. GPC3 influence several central signaling pathways in hepatocellular carcinoma (Kolluri and Ho, 2019) and is also evaluated for CTC capture as described later. GPC3 and other glypicans are GPI-anchored and known to carry HS chains (Filmus et al., 2008; Yoneda et al., 2012), but were also demonstrated to carry CS chains (Chen and Lander, 2001; Toledo et al., 2020). Their GAG chains are located close to the cell membrane due to their proximity to the C-terminus, which is thought to be critical for their interaction with surface receptors (Filmus et al., 2008). GPC3 overexpression increased cell proliferation *in vitro* and *in vivo* for liver cancer cells by enhancing Wnt signaling (Figure 3) (Capurro et al., 2005). Mutagenesis of the GAG attachment site in GPC3 revealed that the HS GAGs were not essential for binding of Wnt ligands (Capurro et al., 2005). Supporting this, the Wnt binding site on GPC3 has recently been located to a hydrophobic groove, which works independently of GAG chains (Li et al., 2019). However, the GAG chains of GPC3 are essential for direct interaction with the Wnt receptors, the Frizzled proteins (Capurro et al., 2014). Upon Wnt stimulation a ternary complex is formed and endocytosed (Capurro et al., 2014). Generally, endocytosis of Wnt signaling complexes seems to be important for canonical Wnt signaling with final stabilization and nuclear accumulation of β-catenin and subsequent gene expression changes (Brunt and Scholpp, 2018). In addition, this signaling axis could be a potential therapeutic target for hepatocellular carcinoma based on a monoclonal antibody recognizing the HS chains of GPC3 (Gao et al., 2014). Overall, it was suggested that GPC3 works as a bridging protein between Wnt and its receptor thereby inducing cell proliferation (Li et al., 2019). The exact interaction dependencies could rely on the expression levels of all three partners (Wnt ligands, Wnt receptors, and GPC3) (Li et al., 2019).

However, proteoglycans can also act as negative regulators of cancer biology. One example for this is decorin, which is modified with a single CS or DS side chain. Decorin can act as an inhibitor of cell proliferation by hampering growth signaling. This repression is thought to occur through growth factor sequestering as well as receptor internalization and degradation, mediated by binding to the decorin core protein (Jarvinen and Prince, 2015). For example, *de novo* expression of decorin in breast cancer cell lines suppressed proliferation and anchorage-independent growth (Santra et al., 2000). Consistently, 30% of decorin-knockout mice formed spontaneous intestinal tumors (Bi et al., 2008), highlighting its potential role as tumor suppressor.

To sum up, proteoglycans appear to have a multi-facetted and important role in cancer cell proliferation by diverse mechanisms, which can vary across different cancer types. When CTCs reach the metastatic site, they often go into an inactive dormancy state (Sosa et al., 2014). Reactivation of cell proliferation is therefore an important factor for establishment of clinically relevant metastatic lesions, in which proteoglycans are actively involved (Elgundi et al., 2019) and which will also be discussed later in more detail.

### Angiogenesis in Cancer

Oxygen supply is essential for cells and their metabolism. *Ex vivo* measurement on xenografts revealed that oxygen perfuses only to around 100 μm deep into the tumor tissue (Olive et al., 1992). Therefore, cancer cells must secure sustained blood supply at an early stage, which can happen by different mechanisms (Xu et al., 2016; Lugano et al., 2020). Several proteoglycans are involved in the complex process of tumor angiogenesis (Iozzo and Sanderson, 2011; Chiodelli et al., 2015). Interestingly, increased vascularization could be observed already in premalignant lesions (Menakuru et al., 2008), possibly explaining how CTCs can be shed already from early stage cancers (Husemann et al., 2008; Stott et al., 2010; Rhim et al., 2012; Zhang et al., 2014; Tsai et al., 2016; Murlidhar et al., 2017). Studies on early cancer cell dissemination are of high clinical importance as it enables the use of CTCs in screening programs for early cancer detection.

One central molecule for angiogenesis is the vascular endothelial growth factor (VEGF) (Ferrara et al., 2003), which has been linked to different proteoglycans as for example biglycan. Cancer cells have been shown to overexpress biglycan (Zhu et al., 2013; Hu et al., 2014; Andrlova et al., 2017; Jacobsen et al., 2017), which has two potential GAG attachment sites carrying either CS or DS chains (Valiyaveettil et al., 2004). Interestingly, biglycan is a homolog of decorin (Fisher et al., 1989), but seems to have tumor promoting capacities by angiogenesis induction in contrast to decorin (Schaefer et al., 2017). Indeed, elevated biglycan levels induced higher density of blood vessels and increased tumor growth *in vivo* of colorectal cancer xenografts via induction of VEGF expression (Hu et al., 2016). In endothelial cells, biglycan binds to Toll-like receptor 2 (TLR2) and TLR4 with activation of the transcription factor family nuclear factor-κB (NFκB). This subsequently leads to increased levels of hypoxia-inducible factor 1-alpha (HIF1α), which drives VEGF expression (Hu et al., 2016) and could finally lead to tumor angiogenesis. VEGF can potentially regulate expression of another proteoglycan linked to cancer and angiogenesis, namely endocan (Grigoriu et al., 2006; Rennel et al., 2007; Roudnicky et al., 2013). Indeed, endocan was detected in the tumor vasculature (Maurage et al., 2009; Roudnicky et al., 2013) as well as in cancer cells (Rennel et al., 2007; Maurage et al., 2009; Xu et al., 2019). Interestingly, it was suggested by Rocha et al. (2014) that endocan binding replaced VEGF from fibronectin in the ECM, creating a positive feedback loop. In head and neck cancer, endocan was strongly co-expressed with angiopoietin-2 (Xu et al., 2019), which can regulate vascular permeability during intra- and extravasation processes (Garcia-Roman and Zentella-Dehesa, 2013) and potentially affect the dissemination of CTCs during metastasis. Importantly, endocan expression is associated with poor survival rate and might also be used as serum biomarker in cancer patients (Grigoriu et al., 2006; Roudnicky et al., 2013; Kim et al., 2018). Biglycan and endocan are important proteoglycans in angiogenesis and thus tumor progression. However, they might not be ideal candidates for CTC technologies as secreted proteoglycans might not be stable targets for cell analysis.

The neuropilins is another proteoglycan family involved in angiogenesis (Ellis, 2006; Niland and Eble, 2019). Neuropilin-1 helps to bind VEGF to the cell surface and forms a trimeric complex together with VEGF receptor 2 (VEGFR2), which was suggested to act as a potential bridge between cancer cells and endothelial cells (Soker et al., 1998, 2002). Indeed, neuropilin-1 was detected in tumor cells of different cancer entities as well as in endothelial cells of the tumor vasculature (Jubb et al., 2012). Overexpression of neuropilin-1 increased xenograft growth (Miao et al., 2000; Hu et al., 2007). Neuropilin-1 carries a single GAG chain of HS or CS, dependent on the cell type (Shintani et al., 2006; Frankel et al., 2008). However, the exact role of the GAG chain is not fully understood. Mutagenesis of the GAG attachment site in neuropilin-1 increased glioma cell invasion (Frankel et al., 2008). Interestingly, global removal of CS by chondroitinase ABC enzyme treatment led to decreased invasion in the same cells. Neuropilin-1 is also physiologically expressed as a GAG-deficient splice variant (namely NRP1-Δ7), which attributes to 10–30% of total neuropilin-1 transcripts depending on cell type or tissue (Hendricks et al., 2016). Importantly, NRP1-Δ7 acted anti-tumorigenic and diminished tumor vascularization in prostate cancer xenografts *in vivo* (Hendricks et al., 2016). Moreover, soluble neuropilin-1 isoforms with anti-tumorigenic functions have been described, which block VEGFR signaling (Gagnon et al., 2000; Cackowski et al., 2004). Overexpression of soluble neuropilin-1 in cancer cells led to disturbed tumor vascularization and cancer cell apoptosis in xenografts (Gagnon et al., 2000). Overall, proteoglycans are connected to tumor angiogenesis and to VEGF signaling with various effects.

### Epithelial-to-Mesenchymal Transition, Migration, and Intravasation

Another important milestone for cancer cells is to gain migratory capacities to leave the primary tumor and invade the surrounding tissue. During the gastrulation phase of embryogenesis, epithelial-to-mesenchymal transition (EMT) causes stationary epithelial cells to undergo major changes into motile mesenchymal-like cells in order to form new germ layers. Molecular changes in transcription factor networks and gene expression facilitate the loss of cell polarity and cytoskeletal reorganization, resulting in an increased migratory capacity (Lim and Thiery, 2012; Lamouille et al., 2014). Cancer cells imitate this developmental EMT program and several studies suggest that proteoglycans are actively involved in this part of cancer progression, thus supporting the relevance of proteoglycans as targets for CTC capture. Situated in the glycocalyx of cancer cells, proteoglycans provide a contact link between the cell membrane and the surrounding ECM, thereby playing a central role in regulating cancer cell adhesion and migration. Some proteoglycans are downregulated in order to enable detachment from the basement membrane facilitating invasion, others are shed from the surface as a different mode of regulation, and some maintain their function throughout the invasive phase. Importantly, the current standard for CTC isolation is based on antibodies against epithelial cell adhesion molecule (EpCAM), which is often downregulated during EMT (Gorges et al., 2012; Hyun et al., 2016). Thus, understanding the process of EMT in terms of proteoglycan regulation is important for their evaluation as alternative CTC-target candidates.

One important modulator of EMT processes is transforming growth factor β (TGFβ), which is known to drive progression of late state malignancies by promoting invasion (Akhurst and Derynck, 2001; Xu et al., 2009). Indeed, TGFβ regulates a multitude of genes with potential cancer-specific effects (Ranganathan et al., 2007; Kowli et al., 2013). Several proteoglycans are connected to TGFβ-signaling. An example of this is the expression of HS-carrying syndecan-4, which was positively regulated by TGFβ in lung cancer A549 cells (Toba-Ichihashi et al., 2016). Expression of this proteoglycan further induced upregulation of the EMT transcription factor zinc finger protein SNAI1 (sometimes referred to as snail) (Toba-Ichihashi et al., 2016), thereby fueling the migratory behavior. This is somewhat surprising, since syndecan-4 plays a well-established role in focal adhesion sites together with integrins, thereby promoting the adhesive phenotype of cancer cells (Echtermeyer et al., 1999; Saoncella et al., 1999).

**FIGURE 4:**
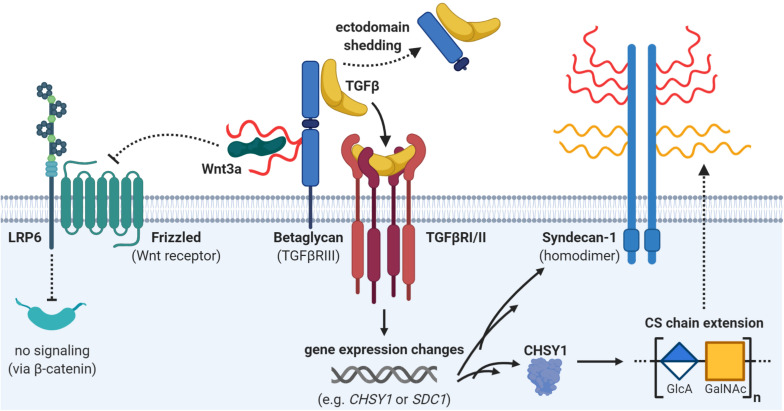
Interplay of proteoglycans and transforming growth factor beta (TGFβ) signaling. The proteoglycan betaglycan is a co-receptor for TGFβ and brings it to the TGFβ receptor (heterodimer of TGFβRI and TGFβRII). However, ectodomain shedding of betaglycan might attenuate TGFβ signaling. Furthermore, betaglycan also dampens Wnt signaling by sequestering Wnt ligands with its heparan sulfate (HS) side chains (in red). Active TGFβ signaling can affect the expression of many different genes such as *SDC1 or CHSY1*. Upregulation of the enzyme chondroitin sulfate synthase 1 (CHSY1) can potentially lead to elongation of chondroitin sulfate (CS) chains on proteoglycans. Indeed, TGFβ can also upregulate one potential CHSY1 targets, namely syndecan-1, which can carry both chondroitin sulfate (CS; in yellow) or heparan sulfate (HS; in red). Details and references are provided in the main text.

Syndecan-1 can also be affected by TGFβ and was suggested as a poor prognostic factor in breast cancer (Hayashida et al., 2006; Nikitovic et al., 2014). Incubation of mouse mammary epithelial cells with TGFβ changed the GAG composition of syndecan-1 from being mainly HS modified to carry nearly equal amounts of HS and CS (Rapraeger, 1989). Notably, increased CS display was not only mediated by attachment of more GAG chains, but also by increased length of individual CS chains (Figure 4). This is in line with a later study showing that TGFβ induced expression of CS synthase 1, a key enzyme involved in the elongation of CS and DS GAG chains (Hu et al., 2015). In addition, other GAG polymer-modifying enzymes have been shown to be transcriptionally affected by TGFβ treatment (Tiedemann et al., 2005; Mohamed et al., 2019), suggesting a role of specific GAG modifications on proteoglycans in regulating the cellular response toward this cytokine.

TGFβ signaling is mediated through heteromeric complex formation of type I and type II receptors (Weiss and Attisano, 2013). However, co-receptors like type III TGFβ receptor, also known as betaglycan, can modulate ligand presentation to the type II receptor (Figure 4). Betaglycan is a cell membrane proteoglycan which may carry both CS and HS GAG chains (Cheifetz et al., 1988). Sulfated HS-modifications on betaglycan have been proven to sequester the Wnt3a ligand and thereby inhibiting proliferation by dampening Wnt signaling (Jenkins et al., 2016). In contrast, TGFβ binding is mediated by the protein core of the proteoglycan, and is therefore insensitive to point mutations disrupting the GAG-attachment sites (Lopez-Casillas et al., 1994). As any other membrane proteoglycan, betaglycan can undergo ectodomain shedding (Weiss and Attisano, 2013). A soluble form of the receptor was shown to result in reduced ligand availability due to its high-affinity interaction with TGFβ and thus decreased TGFβ signaling (Elderbroom et al., 2014). In line with this, it was shown that increased betaglycan expression decreased the invasive behavior of breast cancer cells *in vitro* in response to TGFβ stimulation and that this effect was abrogated when betaglycan was expressed in a shedding-impaired mutant form (Elderbroom et al., 2014). Importantly, metastatic lesions showed lower betaglycan expression compared to matched primary tumors (Hempel et al., 2007). In ovarian cancer cell lines this seems to be mediated through epigenetic silencing, as expression was restored upon epigenetic-acting drugs (Hempel et al., 2007). This indicates that betaglycan might be involved in the dissemination processes and thus the investigation of its biological function in CTCs and metastasis formation would be interesting.

**FIGURE 5:**
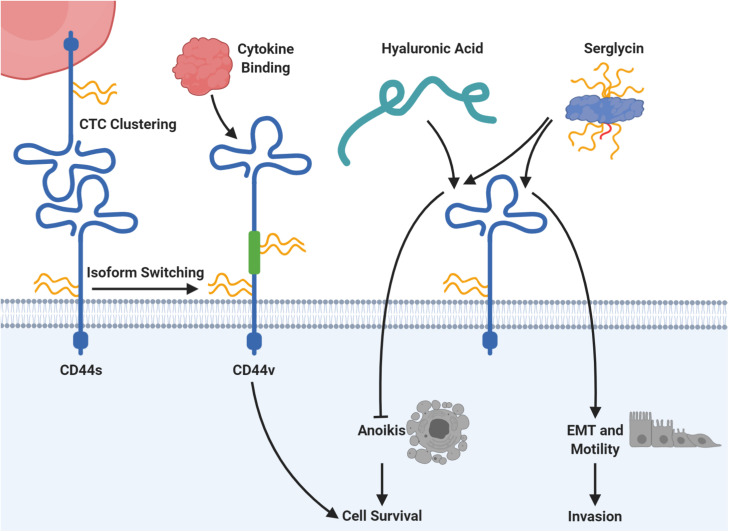
CD44 is connected to several mechanisms of circulating tumor cell (CTC) survival and plasticity. The protein core of CD44 can carry chondroitin sulfate (CS; in yellow), heparan sulfate (HS; in red), or dermatan sulfate (DS; not shown). Homophilic CD44 interactions allow clustering of CTCs, which supports seeding and colonization at the metastatic site in mice. Moreover, expression of the standard CD44 protein form (CD44s) can be switched to expression of CD44 variants (CD44v), which include additional exons via alternative splicing (depicted in green). Those CD44v forms may carry additional glycosaminoglycan chains in dependency of the included exons and promote binding of cancer-related cytokines with downstream signaling for cell survival. The proteoglycan serglycin can carry eight glycosaminoglycan chains of heparan sulfate (HS; in red) or chondroitin sulfate (CS; in yellow) and binds to CD44. Serglycin binding to CD44 induces epithelial-mesenchymal transition (EMT) as well as cell motility and has been proven to be dependent on its CS chains. Moreover, the serglycin-CD44 interaction prevents the induction of anoikis, a specialized form of apoptosis. For this, serglycin competes with hyaluronic acid, which has the same survival promoting effect. Please refer to the specific subsections for details and references.

In addition, proteoglycans might affect cancer cell migration independently of TGFβ signaling. For example, a number of studies have demonstrated a role of serglycin in malignant transformation as described below. Serglycin can carry up to eight CS or HS chains (Kolset and Tveit, 2008) and is widely expressed by hematopoietic cells as well as embryonic stem cells, where it serves functions in storage of intracellular granules and secretion of inflammatory mediators (Toyama-Sorimachi et al., 1995; Schick et al., 2001; Abrink et al., 2004). Elevated serglycin expression was reported for cancer cells in patient tissues and has been linked to aggressive cancer cell phenotypes *in vitro* (Korpetinou et al., 2015). Further, serglycin was identified as an unfavorable prognostic factor in patients suffering from a range of cancers, including glioblastoma (Roy et al., 2017), liver (He et al., 2013), lung (Guo et al., 2017), and nasopharynx (Li et al., 2011). Secreted serglycin from cancer cells was shown to be primarily CS-modified, and transgenic expression of serglycin lacking the GAG attachment site led to decreased migratory capacity of breast cancer cells *in vitro* (Korpetinou et al., 2013). This observed GAG-dependency was further supported by another study focused on lung cancer cells (Guo et al., 2017). Here it was demonstrated that serglycin exerts its functional role during migration by binding to the cluster of differentiation 44 antigen (CD44) with downstream activation of EMT (Figure 5). Blocking of the post-translational CS modification on serglycin abrogated the effect on motility (Guo et al., 2020). CD44 itself is a transmembrane proteoglycan expressed by various cell types (Goodison et al., 1999; Gronthos et al., 2001; Domev et al., 2012) and is normally involved in hematopoiesis, inflammation, and wound healing (Johnson et al., 2000; Dimitroff et al., 2001b; Cichy and Puré, 2003). CD44 is also involved in several important steps during metastasis formation and has been explored as a CTC target in several studies as described later.

When cells gain migratory and invasive capacity, this is often associated with increased remodeling and breakdown of the ECM, which finally enables breaching of the endothelial basement membrane and intravasation into the blood circulation (Eccles, 1999). One contributing factor is the secretion of different proteases by cancer cells, including for example matrix metalloproteinases (MMPs) (Lynch and Matrisian, 2002). MMPs are produced in a catalytically inactive form, which requires proteolytic cleavage for activation. Interestingly, CSPG4 has been shown to facilitate assembly of a ternary complex consisting of pro-MMP2, MMP-cleaving enzyme (MT3-MMP), and the proteoglycan itself at the cell surface of melanoma cells, leading to cleavage and thus activation of MMP2 (Iida et al., 2007). While the interaction with MT3-MMP was shown to be mediated through the core protein of CSPG4, the association with pro-MMP2 was depending on the CS-modification (Iida et al., 2007).

Another enzyme playing an important role in accessing the blood circulation is heparanase, which cleaves HS polysaccharides located in the basement membrane and on the cancer cell surface, leading to increased invasive behavior of cancers (Sanderson et al., 2017; Masola et al., 2018; Elgundi et al., 2019). Several studies have provided evidence that heparanase plays a major role in progression of a variety of cancers including liver cancer (Koliopanos et al., 2001), sarcomas (Cassinelli and Lanzi, 2020), ovarian cancer (Zhang et al., 2013), breast cancer (Maxhimer et al., 2005), and colon cancer (Nobuhisa et al., 2005). In addition to this, it has recently been shown that overexpression of heparanase promotes the formation of cell clusters in MDA-MB-231 breast cancer cells, most likely by modulating the level of intercellular adhesion molecule 1 (ICAM-1) and phosphorylation status of downstream kinases (Wei et al., 2018). Furthermore, the enhanced ability to form clusters correlated with increased number of metastatic foci in the lungs upon tail-vein injection into mice (Wei et al., 2018). Together this data strongly suggests that invasion and intravasation are mediated through HS degradation in the ECM and possibly at the cell surface, leaving behind HS-associated core proteins without this modification.

### Surviving the Journey Through Circulation

For normal cells, detachment from the extracellular matrix leads to cell death through a mechanism of detachment-induced apoptosis, called anoikis (Strilic and Offermanns, 2017). Therefore, CTCs must overcome this major challenge in order to survive in circulation. Studies have pointed toward several cellular strategies to circumvent apoptotic signaling, some involving proteoglycans as important players.

Increased syndecan-4 and heparanase expression have been reported in anoikis-resistant rat endothelial cells (Carneiro et al., 2014). Studying these cell lines, Carneiro et al. (2014) also detected increased level of HS in the culture medium, whereas cell lysates contained increased levels of CS. As another example of proteoglycan involvement in anoikis resistance, overexpression of serglycin in a lung cancer cell line led to increased survival in an anchorage-independent growth assay (Guo et al., 2017). This effect was dependent on CD44 expression. In line with this, increased CD44 expression caused by EMT induction also led to anoikis resistance in immortalized human mammary epithelial cells (Cieply et al., 2015). However, in this study the ability of anchorage-independent growth relied on the hyaluronan-binding capacity of CD44. Interestingly, an early study indicated that serglycin and hyaluronan were competing for the binding to CD44 (Figure 5) (Toyama-Sorimachi et al., 1995). The prominent role of CD44 in escaping anoikis was further strengthened by a study linking the CD44-expressing subsets of two hepatocellular carcinoma cell lines to anoikis resistance (Okabe et al., 2014). Importantly, this study also observed increased CD44 expression in CTCs compared to patient-matched primary tumor biopsies, again highlighting a potential role of CD44 in CTC analysis.

The ability of the cancer cells to establish an immunosuppressive microenvironment, and thereby escape elimination by the immune system, is considered one of the hallmarks of cancer (Hanahan and Weinberg, 2011). Leaving the primary tumor site and entering the hostile environment of the blood circulation further elevate the requirement for immune cell evasion. One specific strategy for suppressing an immune attack is upregulation of CD47, which constitutes an anti-phagocytotic “do-not-eat-me”-signal on the surface of cancer cells (Jaiswal et al., 2009). CD47 is a proteoglycan carrying both CS and HS, and is widely expressed on white blood cells, where it functions as a receptor for thrombospondin-1 in a GAG-dependent manner (Kaur et al., 2011). Interestingly, CD47 expression was upregulated in colorectal CTCs compared to corresponding primary tumor tissue (Steinert et al., 2014). In addition, Baccelli et al. (2013) characterized the metastasis-initiating subpopulation of breast cancer CTCs as positive for EpCAM, tyrosine-protein kinase Met (cMet), CD44, and CD47 (Baccelli et al., 2013). Another study found that blocking of CD47 on 4T1 mouse breast cancer cells prior to tail vein injection significantly reduced the number of lung metastases in mice (Lian et al., 2019).

Another proteoglycan-based mechanism was shown to provide a strategy to avoid the secretion of lytic granules from NK cells, which would be lethal to the cancer cells. Baccelli et al. (2013) demonstrated that expression of telomeric repeat-binding factor 2 (TRF2) controlled a cell-extrinsic pathway, involving upregulation of HS glucosamine 3-*O*-sulfotransferase 4 (HS3ST4), thereby dampening immune surveillance by NK cells (Biroccio et al., 2013). Further, it was revealed that TRF2 overexpression led to upregulated expression of two HS-carrying proteoglycans, glypican-6 and versican, both of which were shown to decrease NK cell degranulation (Cherfils-Vicini et al., 2019). However, whether TRF2 and associated changes in HSPGs play a role for CTC survival in the circulation still needs to be shown.

### Extravasation and Colonization at the Metastatic Site

To form metastatic lesions, CTCs must extravasate and enter the distal tissue. This crucial step in the metastatic cascade is highly inefficient, as the vast majority of CTCs undergo apoptosis, and only a small fraction of the surviving cells succeed in forming metastatic colonies (Massague and Obenauf, 2016; Rejniak, 2016). During extravasation, CTCs adhere to and cross the vascular endothelium in the process of transendothelial migration (TEM) (Reymond et al., 2013). Indeed, multiple factors influence cancer cell extravasation. For instance, capillaries lined with fenestrated endothelial cells and a discontinuous basal membrane in the liver and bone marrow facilitate CTC invasion (Aird, 2007; Strilic and Offermanns, 2017) and contribute to the high incidence of bone and liver metastases (Nguyen et al., 2009; Budczies et al., 2015).

There is ample evidence that CTCs exert what has become known as “leukocyte mimicry,” since many of the adhesion and TEM mechanisms are shared with leukocytes (Strell and Entschladen, 2008). Especially, the selectin family of adhesion molecules, important for hematopoietic progenitor cell homing to the bone marrow, have been implicated in cancer cell extravasation (Dimitroff et al., 2001a, 2004). This is mainly mediated by sialofucosylated carbohydrate ligands, particularly the sialyl Lewis (sLe^X^) structures, which are primarily found on leukocytes (Fukuda et al., 1999) as well as on various cancer cells (Majuri et al., 1995; Renkonen et al., 1997). A particular sialofucosylated glycoform of the proteoglycan CD44 termed hematopoietic cell E-/L-selectin ligand (HCELL) mediates selectin tethering (Sackstein, 2004). E-selectin is highly expressed on endothelial cells in the bone marrow (Burdick et al., 2012). In cooperation with carcinoembryonic antigen, HCELL facilitates cancer cell rolling through binding to E-selectin (Hanley et al., 2006; Thomas et al., 2008), strongly supporting the hypothesis of HCELL-mediated CTC arrest in the vasculature, a crucial step in CTC extravasation (Lee et al., 2014). Interestingly, studies have investigated the presence of over-sulfated GAGs as alternative ligands for selectins in cancer extravasation mechanisms (Martinez et al., 2013), highlighting the importance of altered display of GAGs.

Besides robust adhesion of CTCs to the endothelial wall, CTC clusters also seem to be important for metastatic seeding and outgrowth. In the circulation, CTCs can exist as single cancer cells or as clusters of cancer cells. The prevalence of polyclonal CTC clusters correlates with poor prognosis in patients, and is believed to be an important component for metastatic success (Aceto et al., 2014; Gundem et al., 2015; Cheung et al., 2016). In a recent study, CD44 was identified as a key component in clustering of cancer cells both in patient-derived xenograft (PDX) models in mice and in metastatic breast cancer patients (Liu et al., 2019). Mechanistically, CD44 formed homophilic interactions independent of HA on the cancer cell surface (Figure 5), which in turn triggered activation of a serine/threonine-protein kinase 2 (PAK2) and focal adhesion kinase (FAK) dependent signaling cascade. Knockout of CD44 resulted in loss of CTC cluster formation and reduced lung colonization and metastasis in PDX models.

CD44 is a multi-functional proteoglycan for colonization and priming of the metastatic niche (Zoller, 2011). The standard CD44 (CD44s) comprises exons 1–5 and 16–20, while the splice variants (CD44v) also include various combinations of exons 7–15, whereas exon 6 is missing in humans (Naor et al., 2002). In several cancers isoform switching via alternative splicing of *CD44* is frequently observed (Johnson et al., 2000). For example, CD44v3, CD44v6, and CD44v10 have been implicated in cancer and are the only CD44 isoforms that contain binding sites for cancer-related cytokines and chemokines (Chen C. et al., 2018; Wang Z. et al., 2018). In colorectal cancer, CD44v6 positive cells are able to form metastatic lesions in the liver and lung through interaction with osteopontin (Huang et al., 2012). Importantly, the CD44 protein core can carry HS, CS, KS, or DS, but the GAG content is highly dependent on the isoform and exons involved (Bennett et al., 1995; Greenfield et al., 1999; Clark et al., 2004). Furthermore, cytokines secreted in the tumor microenvironment (e.g., hepatocyte growth factor and stromal-derived factor 1a), increased CD44v6 expression, and assisted colorectal cancer stem cells in colonization and survival through activation of the phosphatidylinositol 3-kinase-protein kinase B (PI3K-AKT) pathway (Todaro et al., 2014).

Once the CTCs have managed to extravasate into the tissue, the nature of the ECM at the secondary site dictates whether the disseminated cancer cells will proliferate into overt metastases, enter a dormant state, or undergo apoptosis (Ghajar et al., 2013; Sosa et al., 2014; Peinado et al., 2017; Goddard et al., 2018). One way in which cancers prime the pre-metastatic niche is through exosome secretion, which facilitates organ-specific engraftment of cancer cells (Simons and Raposo, 2009; Peinado et al., 2012; Hoshino et al., 2015). Interestingly, HS has been shown to play a role in the syndecan-1 mediated formation of the syntenin-ALG2-interacting protein X (ALIX) complex (Baietti et al., 2012; Thompson et al., 2013; Roucourt et al., 2015). Following vesicular release, HSPGs also take part in exosome docking and delivery of vesicular cargo to the recipient cell. This dual role of HSPGs in exosome-mediated crosstalk between cells is fostered by fibronectin that interacts with HS displayed on the surface of exosomes and functions as a heparan sulfate/HS-binding ligand on target cells (Purushothaman et al., 2016; Colombo et al., 2019). Another study reported a correlation between the expression of glypican-1 on the exosomal surface and the tumor burden in pancreatic cancer patients (Melo et al., 2015), supporting a prognostic value of proteoglycans associated with exosomes in carcinogenesis.

At the metastatic site, proteoglycans also contribute by promoting cancer cell engraftment and colonization (Fares et al., 2020). The potential role of serglycin in metastatic dissemination has been investigated in a mouse model of breast cancer, where knockout of serglycin resulted in CTCs unable to establish metastatic tumors although not affecting primary tumor formation (Roy et al., 2016). Correspondingly, increased serglycin expression was shown to facilitate liver colonization by cancer cells in a patient-derived xenograft model of non-small-cell lung cancer (NSCLC) (Guo et al., 2017) as well as to promote hepatocellular carcinoma metastasis to the bone (He et al., 2014).

In summary, proteoglycans are connected to all steps of the metastatic cascade. Notably, some proteoglycans appear to play active roles in several aspects of cancer progression, highlighting these as potential key players of the cancer cell surface. One such proteoglycan is CD44, which is highly involved in EMT, helps to prevent anoikis due to its HA-receptor function, and furthermore takes actions in generating CTC clusters and extravasation, thereby enabling a successful arrival at the metastatic site (Figure 5). Another key proteoglycan seems to be CSPG4, with important roles for the regulation of cancer cell growth and invasion. Furthermore, the studies on CSPG4 presented here demonstrate how transmembrane proteoglycans possess multiple modes of action by engaging with other receptors or signaling molecules through either their cytoplasmic domain, ectodomains, or their GAG chains. With this central role in metastasis and CTC biology, proteoglycans could be an interesting target for CTC technologies. Indeed, proteoglycans are already studied and partly utilized for CTC identification and capture by different technologies. The following section will hence provide a detailed overview on proteoglycans as CTC targets.

## Proteoglycans in Circulating Tumor Cell Diagnostics

Circulating tumor cell detection assays have spurred increasing clinical interest since the prognostic value in progression-free and overall survival was established in patients with metastatic colorectal (Cohen et al., 2009), breast (Cristofanilli et al., 2004), prostate (de Bono et al., 2008), and lung (Krebs et al., 2011) cancer. CTC enumeration from patient blood samples has also demonstrated clinical relevance for several other cancer types such as pancreatic cancer (Kurihara et al., 2008; Bidard et al., 2013; Effenberger et al., 2018) or hepatocellular carcinoma (Schulze et al., 2013; Qi et al., 2018). The presence of detectable levels of CTCs in the peripheral blood is associated with the metastatic capacity of the disease (Allard et al., 2004; Cristofanilli et al., 2004). However, low levels of CTCs have been reported in non-metastatic disease for several cancer indications before and after surgery (Thorsteinsson et al., 2011; Franken et al., 2012; Gazzaniga et al., 2013). Additional studies suggest that CTCs are even shed from premalignant lesions and this opens the possibility for using CTC detection for early diagnosis of cancer (Husemann et al., 2008; Stott et al., 2010; Rhim et al., 2012; Zhang et al., 2014; Tsai et al., 2016; Murlidhar et al., 2017). CTC assays might also have potential as a tool for predicting treatment efficacy and monitoring disease (Schochter et al., 2019; Yang et al., 2019), thereby providing real-time, non-invasive information about the disease by liquid biopsies. Furthermore, many CTC assays do not only enable enumeration but also downstream analyses such as genomic, transcriptomic, proteomic, or phenotypic characterization of cancer cells. Therefore, studying CTCs can also bring novel insight into aspects of metastasis formation, which are still not fully understood (Chaffer and Weinberg, 2011). Despite the interest and potential in analyzing CTCs, the methods are rarely implemented in the clinical setting, as CTC identification requires highly specific markers and an extreme assay sensitivity. Many CTC methods struggle to reach the needed sensitivity, as it is a technical challenge to detect few cancer cells in billions of normal blood cells.

Several CTC enrichment technologies ranging in complexity have been developed (Kowalik et al., 2017; Dianat-Moghadam et al., 2020). From whole blood, CTCs can be enriched along with leukocytes by density fractionation or a simple lysis of the erythrocytes. The crude cell enrichment can be analyzed by direct antibody staining and examined by, e.g., microscopy (Hillig et al., 2015; Werner et al., 2015) or flow cytometry (Hristozova et al., 2012; Watanabe et al., 2014; Lopresti et al., 2019). Because of the rarity of the CTCs, an additional cancer cell-specific enrichment step is, however, often preferred. CTCs can be enriched from the leukocytes based on distinct biophysical properties such as size, density, deformability, or charge (Liu et al., 2015; Mitchell et al., 2015; Shaw Bagnall et al., 2015). Following CTC enrichment, the detection of CTCs will still rely on a staining step, distinguishing for example cytokeratin (CK)-positive CTCs from the remaining CD45-positive leukocytes. Other systems for CTC isolation use cancer- or tissue-specific antibodies to enrich for CTCs (Coumans and Terstappen, 2015) or even leukocyte cell surface proteins like CD45 to deplete for leukocytes (Ozkumur et al., 2013; Karabacak et al., 2014). The positive selection of CTCs is evidently very dependent on highly specific cancer or tissue markers. In order to demonstrate high potential for clinical application, extensive validation of CTC capture platforms must reveal robust clinical sensitivity and specificity (Parkinson et al., 2012). Most pilot studies do not present large-scale clinical data and should hence be interpreted with caution. Inclusion of healthy controls becomes crucial to demonstrate the specificity of the capture and/or detection strategy. Alternatively, some studies apply downstream molecular analyses to verify the tumor origin of detected CTCs, e.g., by mutation detection, which supports the reliability of the CTC assay (Gasch et al., 2013; Muller et al., 2014). From a more technical perspective, pre-analytical conditions such as blood tubes, storage time, and temperature as well as choice of antibody clones can have a huge effect on assay performance (Qin et al., 2014; Ilie et al., 2018; Wu et al., 2020), making comparisons across studies difficult. Furthermore, in the light of exploring proteoglycans as potential CTC targets, the consideration of technical assay parameters become crucial for, e.g., sustaining the GAG stability.

The current clinical standard for CTC enumeration is the CellSearch^®^ platform, which is approved by the American Food and Drug Administration (FDA) for monitoring patients with metastatic breast, colorectal, and prostate cancer. CellSearch^®^ relies on cell enrichment using anti-EpCAM antibody-coated ferrofluid and CTC detection via fluorescent anti-CK antibody labeling (Liberti et al., 2001; Allard et al., 2004; Coumans and Terstappen, 2015). EpCAM-based capture approaches are, however, rarely efficient for epithelial cancers with downregulated EpCAM expression, likely due to EMT, or cancers of mesenchymal origin. Therefore, several studies have been focusing on finding novel markers, which can distinguish EpCAM-low or -negative CTCs from normal blood cells with high specificity and sensitivity to broaden the spectrum of detectable CTC subpopulations (Lampignano et al., 2017; Nicolazzo et al., 2019). As a part of this, multiple strategies using proteoglycans for CTC enrichment or identification are currently under investigation. See Table 1 for an overview on the most used proteoglycans and their applications. Some of them are highlighted in the following.

**TABLE 1:** Proteoglycans used for isolation or identification of clinical circulating tumor cells.

	**Indication**	**Used for**	**Finding**	**References**
Carbonic anhydrase 9 (CA9)	RCC	Immunomagnetic capture	Combined approach with CD147, captured CTCs in 94% of patients. CTC amount correlated with disease stage.	Liu et al. (2016)
		CTC detection (On−chip Sort^®^)	CTCs were detected in 46% of patients (combined CA9 and EpCAM staining).	Naoe et al. (2019)
CD44	BC/TNBC	CTC detection (flow cytometry)	NACT caused significant changes in the quantity of the CTC subsets present in patient blood samples.	Kaigorodova et al. (2018)
		CTC detection (ns)	CTC clusters were associated with poor OS. CTC clusters showed higher CD44 expression.	Liu et al. (2019)
	CRC	CTC detection (flow cytometry	CD133+CD54+CD44+ CTC subset was significantly associated with liver metastasis and had a prognostic value in CRC patients.	Fang et al. (2017)
	Gastric cancer	CTC detection (FACS)	The amount of EpCAM+CD44+ CTCs, but not EpCAM+CD44− CTCs, correlated with disease progression and venous invasion.	Watanabe et al. (2017)
		Negative immunomagnetic enrichment (anti-CD45)	CD44 was a marker for tumorigenic CTCs.	Toyoshima et al. (2015)
	NSCLC	Immunomagnetic capture	CD44+ CTCs were associated with lymphatic invasion and tumor size. CD44+ CTCs were more sensitive to TRAIL-induced apoptosis.	Yan-Bin et al. (2020)
	OSCC	Immunomagnetic capture	CD44+ CTCs showed increased self-renewal capability and chemotherapy-resistance. Clinical correlation between increased CD44v6 and loco-regional aggressiveness and recurrence.	Patel et al. (2016)
Chondroitin sulfate proteoglycan 4 (CSPG4)	Melanoma	Immunomagnetic capture	Analysis of RNA suggested that CSPG4+ CTCs were distinct from CTCs enriched by another melanoma marker, ABCB5.	Aya-Bonilla et al. (2019)
		Immunomagnetic capture	Significant difference between CTC numbers in healthy controls, stage I/II and stage III/IV, using multiple markers. Decrease in CTC numbers during treatments was associated with longer OS and shorter response to treatment.	Freeman et al. (2012), Klinac et al. (2014)
		Immunomagnetic capture (with subsequent depletion of CD45+ cells)	1–20 CTCs found per 5 mL blood and *BRAF* genetic heterogeneity was detected among CTCs.	Sakaizawa et al. (2012)
		Immunomagnetic capture	≥2 CTCs correlated with OS for stage III and IV patients. CTC-positivity correlated with stage.	Ulmer et al. (2004)
		Immunomagnetic capture and IF	MCAM/CSPG4-positive CTCs and *RAS/RAF* mutational status were associated.	Gorges et al. (2019)
		CellSearch^®^ Melanoma Kit	CTC positivity in early-stage disease correlated with OS at 24 months.	Anand et al. (2019)
			Strong correlation between CTC positivity and PFS and OS; and between CTC numbers and ctDNA-levels.	Bidard et al. (2014)
			Prospective study of stage IV patients. Baseline CTC-positivity independently predicted poorer PFS after 180 days.	Hall et al. (2018)
			≥2 CTCs at baseline was an independent prognostic factor for poor OS.	Khoja et al. (2013)
		CellSearch^®^ Melanoma Kit	Prospective study of 243 stage III patients. CTC-positivity at baseline independently predicted poorer relapse-free survival within 6 and 54 months.	Lucci et al. (2020)
			Retrospective study; baseline ≥ 2 CTCs correlated with OS in stage III/IV patients. 95% of healthy subjects had no CTCs.	Rao et al. (2011)
			Difference in CTC positivity for stage I/II vs. IV. Only 2.9% of healthy subject had CTCs.	Roland et al. (2015)
		CTC detection (flow cytometry)	Early-stage CTCs expressed mainly one marker, late-stage CTCs expressed more. 42% of CTCs expressed CSPG4.	Gray et al. (2015)
		CTC detection (flow cytometry)	CTCs were found in 14/22 patients.	Liu et al. (2011)
		CTC detection (IF)	5% of patients had ≥100 CTCs/mL. In these, unique clonal populations were identified.	Ruiz et al. (2015)
		Characterization by Surface-enhanced Raman spectroscopy with αCSPG4	CTC surface marker levels, including CSPG4, were altered during treatment.	Tsao et al. (2018)
C-X-C chemokine receptor type 4 (CXCR4)	HCC	CTC detection (RISH)	CTCs were detected in 89.9% of all patients and CXCR4 expression was associated with different CTC subsets.	Bai et al. (2020)
	NSCLC	CTC detection (flow cytometry)	CXCR4 expression was increased on EpCAM- CTCs.	Yin et al. (2015)
		CTC detection (flow cytometry)	CXCR4+ CTCs showed potential as a predictive marker for OS in NSCLC patients.	Reckamp et al. (2009)
Glypican-3 (GPC3)	HCC	Immunomagnetic capture and CTC detection (flow cytometry)	High GPC3+ CTC amount correlated with shortened disease-free survival in non-metastatic HCC patients.	Hamaoka et al. (2019)
		Immunomagnetic capture	Capture cocktail (together with anti-ASGPR, anti-EpCAM) found higher CTC numbers than each antibody alone.	Court et al. (2018)
		CTC detection (ImageStream^®^)	12.5% of all found CTCs were GPC3+.	Ogle et al. (2016)
	HCC/CCA	CTC detection (IHC)	1 out of 14 patients with CTCs had GPC3+ CTCs.	Nam et al. (2016)
Syndican-1 (SDC1/CD138)	MM	Immunomagnetic capture	CD138+ CTCs strongly correlated with disease burden and treatment response.	Wang et al. (2019)
	MM	Immunomagnetic capture	20–184 CD138+ CTCs detected in patient blood/mL (2–5/mL healthy blood). Patients in remission had fewer CTCs than other patients.	Qasaimeh et al. (2017)

*+, positive; ABCB5, ATP-binding cassette sub-family B member 5; ASGPR, asialoglycoprotein receptor; BC, breast cancer; CA9, carbonic anhydrase 9; CCA, cholangiocarcinoma; CD, cluster of differentiation; CK, cytokeratin; CRC, colorectal cancer; CSPG4, chondroitin sulfate proteoglycan 4; CTC, circulating tumor cell; ctDNA, circulating tumor DNA; CXCR4, C-X-C chemokine receptor type 4; EpCAM, epithelial cell adhesion molecule; FACS, fluorescence-activated cell sorting; GPC3, glypican-3; HCC, hepatocellular carcinoma; IF, immunofluorescence; IHC, immunohistochemistry; ISET, isolation by size of epithelial tumor cells; MCAM, melanoma cell adhesion molecule; MM, multiple myeloma; NACT, neoadjuvant chemotherapy; ns, not specified; OSCC, oral squamous cell carcinoma; OS, overall survival; PFS, progression-free survival; RBC, red blood cell; RCC, renal cell carcinoma; RISH: RNA *in situ* hybridization; SDC1, syndican-1; TNBC, triple-negative breast cancer; TRAIL, TNF-related apoptosis-inducing ligand.*

A well-known example is the CellSearch^®^ Circulating Endothelial Cell Kit, which can be used for the enrichment of circulating melanoma cells that are EpCAM-negative by nature (Rao et al., 2011; Khoja et al., 2013). After capture using ferrofluid coupled with antibodies against melanoma cell adhesion molecule (MCAM), circulating melanoma cells are identified by staining with antibodies against high molecular weight melanoma-associated antigen (HMW-MAA), also known as CSPG4.

As described earlier, CSPG4 has been linked to many aspects of the metastatic cascade, including proliferation, migration, as well as ECM-remodeling and is expressed across many cancer types (Ilieva et al., 2018). Moreover, CSPG4 is expressed in a majority of melanoma lesions (Real et al., 1985) and is a well-characterized surface marker for melanoma (Ilieva et al., 2018). Multiple retrospective studies using the MCAM/CSPG4 CellSearch^®^ Kit have found that CTC levels detected at baseline correlates with overall survival in late-stage melanoma (Rao et al., 2011; Khoja et al., 2013; Bidard et al., 2014) (Table 1). Recently, two large prospective studies also evaluated the prognostic significance of MCAM/CSPG4-positive CTCs in cutaneous melanoma. In a study of 93 stage IV patients, Hall et al. (2018) found that presence of CTCs at baseline was associated with shorter progression-free survival after 6 months compared to CTC-negative patients. Later, the same research group showed that CTC-positivity at baseline for stage III patients (*n* = 243) was an independent predictor of relapse-free survival within 6 and 54 months (Lucci et al., 2020). The CTC levels in these studies were not associated with primary tumor characteristics, such as ulcerations, tumor thickness, and mutational status. Therefore, MCAM/CSPG4-positive CTC numbers may add additional information on top of clinicopathological characteristics for clinicians to foresee the disease course in the future.

Interestingly, CTC-negative melanoma patients have been found to have better progression-free or relapse-free survival compared to CTC-positive patients (Hall et al., 2018; Lucci et al., 2020). However, a significant proportion of late-stage melanoma patients still appear to have no CTCs detectable by CellSearch^®^ (Rao et al., 2011; Roland et al., 2015; Hall et al., 2018). This has also been reported for other CSPG4-based methods (Ulmer et al., 2004; Ruiz et al., 2015) as well as for CSPG4-independent isolation methods (Khoja et al., 2014; Aya-Bonilla et al., 2019). This may simply be due to the rare nature of CTCs. However, there could be CSPG4-negative CTC subpopulations, which are not captured by CSPG4-dependent strategies. In fact, one study found that of 31 melanoma patients with CTCs detectable by other markers, only 42% had CSPG4-positive CTCs (Gray et al., 2015), suggesting a need for multi-marker approaches.

During the past decade, multiple other studies investigated the potential of CSPG4 for CTC capture and/or identification in melanoma (Table 1). Up to 4 CTCs per mL blood was found using CSPG4 immunomagnetic capture (Sakaizawa et al., 2012), which is similar to the reported CTC numbers using the MCAM/CSPG4 CellSearch^®^ kit (Khoja et al., 2013). However, a study by Ruiz et al. (2015) using a CSPG4-based immunofluorescent microscopy approach without prior enrichment step identified a mean of 14.9 CTCs per 1 mL blood samples from melanoma patients (*n* = 40), without potential CTC hits in healthy control samples (*n* = 10). These variations in CSPG4-positive CTCs can be explained to some extent by the use of different CTC enrichment strategies, varying markers for CTC identification or other technical differences in the assays.

Commonly mutated genes in melanoma, such as *BRAF* and *NRAS* (Colombino et al., 2012), are upstream activators of ERK signaling (Savoia et al., 2019). As CSPG4 expression has been connected to ERK signaling (Ampofo et al., 2017), these mutations might be particularly important in CSPG4-positive CTCs. Indeed, a recent study found that CTCs enriched by CSPG4-based method presented more *RAS/RAF* mutated cells compared to CTCs isolated only by physical properties (Gorges et al., 2019). Since some therapeutic approaches target the serine/threonine-protein kinase B-raf (BRAF) (Holderfield et al., 2014), it is possible that the CSPG4 expression might also decrease in response to this form of treatment, which could affect the prospect of using CSPG4 alone to monitor CTC numbers. Indeed, initial longitudinal study of CTC heterogeneity in 10 stage IV melanoma patients suggested that expression of CSPG4 on CTCs may be downregulated in response to BRAF and mitogen-activated protein kinase kinase (MEK)-inhibiting therapy (Tsao et al., 2018). However, to our knowledge, none of the major studies on CSPG4-positive CTCs in cutaneous melanoma have yet found any correlation between CTC levels and BRAF-mutational status or adjuvant therapy.

Another recent study revealed that the transcriptomic profile of CSPG4-enriched CTC populations from six patients was dominated by up-regulation of tumor necrosis factor alpha (TNFα)/nuclear factor kappa B (NF-κB) as well as signal transducer and activator of transcription (STAT) pathways (Aya-Bonilla et al., 2019). Both signaling pathways have central roles for cell proliferation as well as cell survival (Wu and Zhou, 2010; Igelmann et al., 2019). Furthermore, *in silico* analysis found other genes upregulated in the CSPG4-enriched population to be connected to metastasis, tumor growth, and melanoma biology (Aya-Bonilla et al., 2019), which indicates an interesting biological role of CSPG4-positive CTCs for melanoma progression.

Overall, CSPG4 is a relevant surface marker for melanoma CTCs and is hence evaluated in many studies. Although little is still known about the biological role of CSPG4-positive CTCs, they might represent a specific subpopulation. This potential bias should be considered when using only CSPG4 for CTC capture or CTC identification.

CD44 has also been explored for CTC analysis. CD44 is widely expressed (Goodison et al., 1999), and as previously described it acts as a receptor for a variety of ligands. Particularly well-described is the interaction with HA, which constitutes a major part of the glycocalyx and ECM (Banerjee et al., 2016). Upregulation of CD44 confers tumorigenicity, metastatic capacity, and drug resistance to primary tumor cells as well as CTCs (Naor et al., 2002; Fitzgerald and McCubrey, 2014). The abnormal expression of CD44 splice variants is associated with treatment refractoriness, recurrence, and prognosis (Katoh et al., 2015), and overexpression of both CD44s and variants serves a long list of biological functions across many cancer types (Chen C. et al., 2018). Since isoform switching introduces new cancer-related antigens, development of both anti-CD44s and anti-CD44v antibodies has attracted much interest.

As a CTC isolation tool, anti-CD44 antibodies have been used to capture CTCs from cancer patient blood (see Table 1). Yan-Bin et al. (2020) investigated the CD44-positive CTC abundance in NSCLC patients by immunomagnetic enrichment and evaluated the correlation to clinical characteristics. None or very few CD44-positive cells were detected in the 30 controls in contrast to frequent CTC observations in the 128 patient samples. Detected CTCs associated negatively with serum TNF-related apoptosis-inducing ligand (TRAIL) levels, suggesting that CD44-positive CTCs could be more vulnerable to TRAIL-induced apoptosis through death receptor 4 and 5 signaling (Yan-Bin et al., 2020). A small study on gastric cancer patients (*n* = 26) and healthy controls (*n* = 10) associated increased prevalence of EpCAM- and CD44-positive CTCs in patients with tumor depth, disease progression, and venous invasion (Watanabe et al., 2017). Consequently, CD44-based CTC detection was suggested to reflect the malignant potential of the tumor. The authors, however, disregard EpCAM-positive cells found in all healthy controls and the few double positive cells found in 2 healthy controls as either non-specific immunological reactions or contaminating skin cells. Again, this discrepancy highlights the demand for CTC validation, as for example via genomics. Another study analyzed CD44-positive CTCs isolated by immunomagnetic enrichment from 30 oral squamous cell carcinoma (OSCC) patients and 15 healthy controls (Patel et al., 2016). Self-renewal and proliferation capability of the CD44-positive cells were observed by increased sphere-forming capacity unlike the CD44-negative sorted population. Moreover, cisplatin resistance assays confirmed a drug-resistant phenotype associated with the CD44-positive population. This was specifically associated with high transcript levels of *CD44v6*, as opposed to *CD44s*, as well as elevated levels of the stemness marker *NANOG*. Furthermore, the different expression levels strongly correlated with the primary tumor profile and, importantly, clinicopathological parameters such as late-stage, loco-regional aggressiveness, and relapse. The findings suggest that detection of CD44v6-positive CTCs could be used to predict disease progression, therapy outcome, and recurrence.

In addition, CD44 is being evaluated for novel therapeutic approaches against CTCs. For instance, *in vivo* homophilic CD44-mediated CTC clustering of metastatic breast cancer cells in mice was largely inhibited by the administration of anti-CD44 neutralizing antibody, leading to decreased metastatic capacity (Liu et al., 2019). In summary, numerous studies of applications to target CD44-positive CTCs underline its potential in therapy and as a valuable marker for prognosis and treatment response.

Another interesting proteoglycan for clinical purposes is glypican-3 (GPC3), which is upregulated amongst several cancer entities with highest positive case rates in hepatocellular carcinoma (HCC). Importantly, GPC3 has been reported to discriminate between HCC and non-malignant lesions (Zhu et al., 2001; Wang et al., 2008) or other liver-associated cancers like cholangiocarcinoma (CCA) (Man et al., 2005). Nowadays GPC3 is included in a diagnostic HCC panel together with glutamine synthetase and heat shock protein 70, according to guidelines of the European Society for Medical Oncology (ESMO) (Vogel et al., 2019) and the American Association for the Study of Liver Diseases (AASLD) (Marrero et al., 2018). Furthermore, GPC3 might also be used as a serum biomarker (Capurro et al., 2003; Hippo et al., 2004) and is exploited for different targeted cancer therapy approaches (Sawada et al., 2012; Feng et al., 2013).

Several studies have been utilizing GPC3 for analysis of CTCs in HCC patients (Table 1). Anti-GPC3 antibodies have been used for positive immunomagnetic enrichment of CTCs (Court et al., 2018; Hamaoka et al., 2019). Hamaoka et al. (2019) found in a prospective, single-institution study that most of the 85 examined HCC patients had GPC3-positive CTCs with a median of 3 CTCs in 8 mL blood samples, whereas negative controls (in total *n* = 27) such as healthy individuals (*n* = 12) or individuals with inflammatory diseases (*n* = 4), only had a median of 1 GPC3-positive cell in the blood samples (Hamaoka et al., 2019). Moreover, patients with 5 or more CTCs showed shorter disease-free survival compared to patients with fewer GPC3-positive CTCs. Another study by Court et al. (2018) analyzed GPC3 in a capture cocktail together with antibodies against asialoglycoprotein receptor (ASGPR) and EpCAM. Importantly, the combined capture approach with all three targets, isolated higher CTC numbers in patients than each antibody alone. This approach detected CTCs in 96.7% of all HCC patients (*n* = 61) with a median of 6 CTCs per 4 mL blood. In contrast, in healthy controls (*n* = 8) maximum one potential CTC hit was found. Moreover, CTC numbers were increased in more advanced stages compared to early stages. This effect was even more pronounced for the subfraction of vimentin-positive CTCs, which presumably are generated by EMT processes. This highlights the importance of CTC capture strategies independent of potential EMT target proteins like EpCAM, which are often downregulated during EMT. Indeed, varying or low EpCAM expression has already been reported for CTCs originating from other cancer entities (Hyun et al., 2016; de Wit et al., 2018) and should be taken into consideration when designing or interpreting CTC capture assays.

In summary, GPC3 is currently evaluated as a therapeutic target, serum biomarker, and importantly for CTC analyses, where it has been used both for capture (Court et al., 2018; Hamaoka et al., 2019) and identification (Ogle et al., 2016) of CTCs in HCC. Since only few studies have been performed, further studies are needed to prove the feasibility of GPC3 for clinical CTC analyses. Although GPC3 is a well-established diagnostic marker for HCC, further characterization or validation of the potential GPC3-positive CTC hits, for example via molecular analyses, is to our knowledge missing so far.

As described, there is a great diversity among the proteoglycans associated with different cancer types. The different proteoglycans facilitate distinctive processes in the metastatic cascade and their universal expression suggests that proteoglycans are an essential feature for all cancers. The complexity is further expanded when considering the GAG composition. An increasing number of studies indicate that at least some of the functions of proteoglycans are exerted through specific GAG chains. However, only a few studies focused on targeting the GAG part or GAG composition of proteoglycans when isolating CTCs.

**FIGURE 6:**
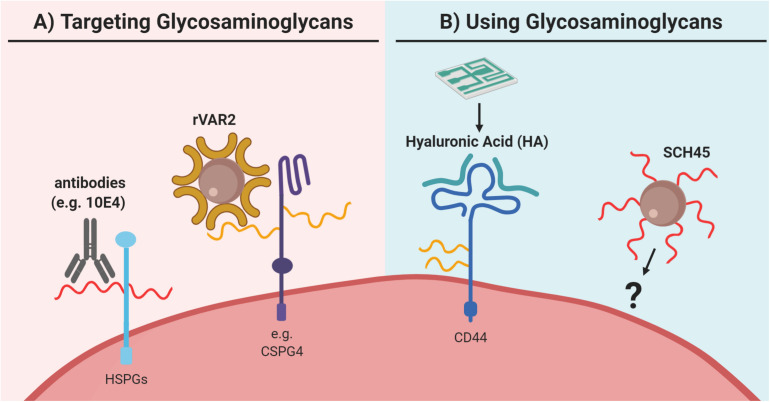
Utilization of glycosaminoglycans for capture of circulating tumor cells (CTCs). **(A)** Glycosaminoglycans can be directly targeted as for example via antibodies like 10E4, which binds to heparan sulfate (HS; in red) of heparan sulfate proteoglycans (HSPGs). However, to our knowledge, this approach has not been explored for CTC capture. Furthermore, for CTC capture the recombinant protein VAR2CSA (rVAR2) can be used, which binds to oncofetal chondroitin sulfate (in yellow) as for example identified on chondroitin sulfate proteoglycan 4 (CSPG4). **(B)** Glycosaminoglycans have been applied in the reversed approach as capture agent for CTC enrichment. Here, glycosaminoglycan-based probes were used to capture CTCs. For example, a microfluidic chip has been coated with hyaluronic acid (HA; in green) to capture CTCs via its interaction with the HA-receptor CD44. Similarly, the heparan sulfate-based probe SCH45 has been coupled to magnetic beads to capture CTCs in hepatocellular carcinoma in a microfluidic setup, but the exact cellular target of SCH45 in these CTCs remains unknown. Generally, both strategies are relatively new for CTC capture and clearly further extensive validation is needed. Please refer to the main text for details and references.

A wide variety of qualitative and quantitative methods has been developed for studying glycocalyx components. As the biosynthesis of glycans is non-template driven and complex, their analysis may often be challenging. Several approaches take advantage of the large repertoire of glycan-binding proteins and antibodies to distinguish between different glycan classes. For large screenings, glycan microarrays have been developed that may probe for different glycan classes or subclasses. This approach has been used to screen breast CTCs for glycan markers, which identified a specific *O*-glycan epitope as a potential target (Wang et al., 2015). Microarrays and cell-based libraries have also been developed to screen for GAG-binding proteins (Rogers and Hsieh-Wilson, 2012) and these may be useful for identifying GAG-based CTC targeting reagents. However, microarrays for detection of cell-surface GAGs, which could be useful for identifying GAG biomarkers on CTCs, have not been constructed. CS and HS-specific antibodies, such as CS56 and 10E4, and GAG-binding proteins, like fibroblast growth factor, are also commonly used in flow cytometry and microscopy-based assays (Figure 6A). These may assess the relative levels of GAGs, however, they do not convey specific structural information due to their low specificity or affinity toward their targets (Yamagata et al., 1987; Smetsers et al., 2004; ten Dam et al., 2007). For this, GAGs will have to be isolated and analyzed, often by chromatography, mass spectrometry, or nuclear magnetic resonance. The structural characterization of GAGs is challenging due to heterogeneity of the polymers. Hence, analysis is often limited to disaccharide analysis, which does not allow for sequencing of intact GAG chains. This is even more technical challenging for CTCs because of the limited input material due to their low abundance. Similarly, while different proteoglycan core proteins can be probed with antibodies, fine structural analysis of their GAG attachment sites is only achieved by glycoproteomic methods. While these analyses may be laborious, they are highly descriptive and may provide novel insight into structural alterations on cancer cells, both on the protein and GAG level. For example, one study found that several major ECM proteoglycans had elevated levels of N-glycosylation in pancreatic cancer tissues (Pan et al., 2014). In addition, another study identified novel CS linkage region modifications in CS glycopeptides from the inter-α-trypsin inhibitor complex, which is abundant in plasma from cancer patients (Gomez Toledo et al., 2015). To our knowledge, glycoproteomics has not been used for analysis of CTCs, and could potentially help identify novel targets.

**FIGURE 7:**
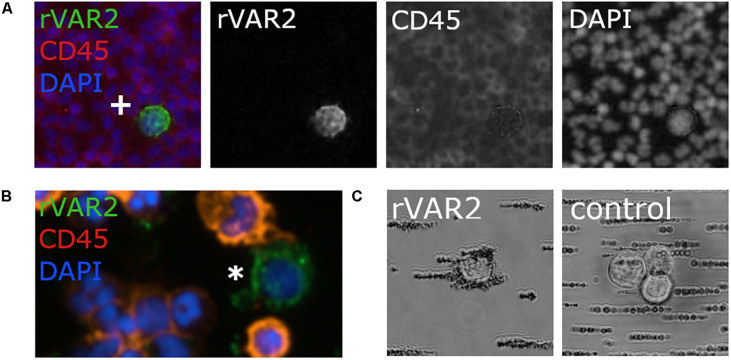
Recombinant VAR2CSA (rVAR2) can be used for staining and capture of potential circulating tumor cells (CTCs). **(A)** Immunofluorescence staining of the colorectal cancer cells COLO205 (marked with cross) with rVAR2 (green), anti-CD45 (in red) to mark normal blood cells, and DAPI (in blue) to mark cell nuclei. **(B)** One potential CTC hit (green by rVAR2 stain; marked with white asterisk) in a blood sample from a colorectal cancer patient with the same staining as described in panel A. **(C)** Magnetic beads coated with rVAR2 bind specifically to COLO205 cells, compared to non-rVAR2 control beads. Pictures were kindly provided by Mette Ø. Agerbæk, Amalie M. Jørgensen, and Nicolai T. Sand.

If succeeding in finding specific binding moieties, the GAG chains would be an alternative novel approach for CTC enrichment or detection. We have previously shown the use of the recombinant VAR2CSA malaria protein (rVAR2) as a novel CTC-targeting reagent (Figures 6A, 7) (Agerbaek et al., 2018; Bang-Christensen et al., 2019; Sand et al., 2020). rVAR2 binds to a distinct type of CS, termed oncofetal CS, expressed by placental as well as cancer cells (Salanti et al., 2015). The native VAR2CSA binds to a specific CS oligosaccharide motif in the placenta during normal physiological conditions (Gowda, 2006; Ayres Pereira et al., 2016; Toledo et al., 2020). A study using a library of cells with knockouts of GAG biosynthesis genes, indicated that 4-*O*-sulfated CS is essential for rVAR2 binding (Chen Y. H. et al., 2018). The specific oncofetal CS-carrying proteoglycans have been examined by screening of rVAR2 binding to more than 3500 cell surface proteins (Salanti et al., 2015) as well as by rVAR2-affinity chromatography coupled to glycoproteomics, using tumor and placenta samples (Toledo et al., 2020). These studies showed that the distinct oncofetal CS is displayed on multiple proteoglycans such as CSPG4 or CD44 in cancer cells, indicating an important function of oncofetal CS in the disease development. Moreover, rVAR2 binds to cancer cells independently of tumor origin and oncofetal CS is expressed both in primary and metastatic lesions (Salanti et al., 2015). This has also been shown in a metastatic murine model, where rVAR2 binding furthermore inhibited integrin signaling and seeding of CTCs (Clausen et al., 2016). As studies have also indicated that rVAR2 binds to cancer cells independent of EMT processes (Agerbaek et al., 2018; Bang-Christensen et al., 2019), oncofetal CS could be an advantageous target for CTC enrichment. In line with this, rVAR2-coated paramagnetic beads have been used to capture CTCs from blood samples of different carcinoma patients (*n* = 44) and glioma patients (*n* = 10) in small proof-of concept studies (Agerbaek et al., 2018; Bang-Christensen et al., 2019). Therefore, the rVAR2-based approach offers an alternative capture approach, demonstrating how GAG-targeting can allow the capture of CTCs independently of single target proteins, like EpCAM.

Actually, the reversed approach can also be utilized for CTC capture (Figure 6B). For example, Gopinathan et al. (2020) coated a synthetic HS-based octasaccharide probe (SCH45) onto magnetic beads, that were used in combination with a microfluidic chip to isolate CTCs from 65 advanced or metastatic cholangiocarcinoma patients. Single CTCs or CTC clusters were detected in all samples with ≥ 1 CTCs per 1 mL blood, even in patients with no distant metastases. Previous comparable CellSearch^®^-based studies found only 17% of CCA patients positive for ≥2 CTCs per 7.5 mL blood (Yang et al., 2016). However, only three healthy controls were included in the HS-based study. Moreover, the authors reported that studies evaluating whether this approach could be employed to capture EpCAM-negative CTCs are currently ongoing (Gopinathan et al., 2020). In addition it would be interesting to identify the binding target of the SCH45-coated beads in order to characterize the captured CTC population.

Another approach exploited the GAG-receptor function of CD44 in order to capture CTCs (Wang M. et al., 2018). Purified HA, the ligand of CD44, was coated to a microfluidic chip and showed 91% retrieval of CD44-overexpressing A549 cells spiked into blood. Also other cancer cell lines from different cancer entities were captured with comparable efficiencies. Although also lacking healthy controls, the study found between 1–18 putative CTCs per 1 mL blood from 9 of 10 NSCLC and 5 of 5 breast cancer patients as detected through CK- and DAPI-staining.

The utilization of GAGs for CTC technologies is a relatively new approach. Most studies have been limited to smaller pilot studies so far and further molecular characterization of the putative CTC hits is needed to prove their cancer-origin and thus the reliability of the CTC assay. Clearly, the establishment of specific GAGs as biomarkers for clinical CTC diagnostics needs extensive validation in large-scale studies in the future. However, GAGs have the potential to capture or identify broader and more heterogenous CTC populations as they are often independent of a single protein and thus might be less prone to gene expression changes associated with different or transient cancer cell phenotypes.

## Concluding Remarks

Circulating tumor cell analyses have the potential to allow prognostic and predictive insights by convenient liquid biopsies. However, novel biomarkers are needed to enable the necessary assay sensitivity and specificity to detect CTCs. Another unsolved problem is that most CTC assays introduce biases in regards to which CTC subpopulations can be captured as they are often based on single biomarkers. Therefore, a CTC capture approach based on a combination of several biomarkers could be beneficial. Another solution for this problem could be the targeting of cancer-specific changes in the GAGs (the “GAGome”), or other known glycocalyx components, which should in principle, allow the capture of more heterogenous CTC populations. Studies on clinical CTCs and their proteoglycans, GAGs, or general glycocalyx structure are still not strongly represented, probably due to associated technical challenges of glycocalyx characterizations. However, structural insights would be beneficial for improving or defining novel CTC capturing strategies based on proteoglycans or their GAGs and to explore whether these strategies then better reflect the heterogenic cancer cell population.

## Author Contributions

TA, SB-C, AJ, CL, CS, NS, and MA conceived and drafted the manuscript. TA designed the figures with scientific input from co-authors. All authors critically revised the manuscript.

## Conflict of Interest

TC, AS, and MA are shareholders in VAR2 Pharmaceuticals holding the intellectual property rights to use rVAR2 for binding oncofetal chondroitin sulfate and to capture circulating tumor cells. VarCT Diagnostics is a subsidiary to VAR2 Pharmaceuticals.

The remaining authors declare that the research was conducted in the absence of any other commercial or financial relationships that could be construed as a potential conflict of interest.
